# Combining Metabolomics and Proteomics to Reveal Key Serum Compounds Related to Canine Intervertebral Disc Herniation

**DOI:** 10.3390/metabo15060396

**Published:** 2025-06-12

**Authors:** Anita Horvatić, Josipa Kuleš, Andrea Gelemanović, Ozren Smolec, Boris Pirkić, Marko Pećin, Ivana Rubić, Vladimir Mrljak, Marko Samardžija, Marija Lipar

**Affiliations:** 1 Faculty of Food Technology and Biotechnology, University of Zagreb, 10000 Zagreb, Croatia; 2 Faculty of Veterinary Medicine, University of Zagreb, 10000 Zagreb, Croatia; ozren.smolec@vef.unizg.hr (O.S.); boris.pirkic@vef.unizg.hr (B.P.); marko.pecin@vef.unizg.hr (M.P.); irubic@vef.unizg.hr (I.R.); vladimir.mrljak@vef.unizg.hr (V.M.); smarko@vef.unizg.hr (M.S.); marija.lipar@vef.unizg.hr (M.L.); 3 Mediterranean Institute for Life Sciences (MedILS), University of Split, 21000 Split, Croatia; agelemanovic@medils.unist.hr

**Keywords:** intervertebral disc herniation, canine serum, metabolomics, TMT, proteomics

## Abstract

**Background/Objectives**: Canine intervertebral disc herniation (IVDH) is an important musculoskeletal pathology. Unlike in humans, IVDH mechanisms in dogs are underinvestigated from a system-level integrative omics point of view. The aim of this study was to identify key serum molecular players in canine IVDH. **Methods**: An integrative multi-omics approach combining high-resolution LC-MS-based untargeted metabolomics and tandem mass tag (TMT)-based proteomics was applied. Additionally, serum zinc concentration was determined by spectrophotometry. **Results**: Nineteen serum metabolites were differentially abundant in IVDH dogs. Metabolite analysis highlighted dysregulation in lipoic acid and branched-chain amino acid (BCAA) metabolism, with elevated levels of valine, leucine, and isoleucine in IVDH. These findings suggest disrupted energy, nitrogen, and neurotransmitter metabolism, potentially contributing to the IVDH pathophysiology. Additionally, lower serum uridine, possibly influenced by BCAA accumulation, was observed, indicating altered neuroinflammatory responses. ELISA validation confirmed elevated serum levels of zinc-α2-glycoprotein (ZAG), alpha-1-microglobulin/bikunin precursor (AMBP), and vitronectin (VTN) in IVDH, supporting immune modulation and neuroprotective mechanisms. Serum prekallikrein (KLKB1) and Protein C inhibitor (SERPINA5), involved in fibrin cloth formation, were found to be lowered in IVDH patients. Pathway enrichment revealed disturbances in aromatic amino acid biosynthesis, with elevated phenylalanine, tyrosine, and tryptophan influencing neurotransmission and inflammation. In addition, elevated serum Zn concentration emphasized its antioxidant importance in immune response, wound healing, and neuropathic pain signaling. **Conclusions**: Integration with our prior CSF multi-omics data reinforced the relevance of identified molecules in IVDH-associated neurodegeneration, inflammation, and repair processes. This study offers insight into potential diagnostic biomarkers and therapeutic targets for canine IVDH through serum-based molecular profiling.

## 1. Introduction

Intervertebral disc degeneration (IVD) is a common multifactorial musculoskeletal phenomenon in dogs and is characterized by the degradation of the extracellular matrix, mainly proteoglycans and collagen. Once the degenerative process has begun, a cascade of events is triggered that ultimately leads to structural failure of the IVD and clinical signs of disease [1]. Large animal models, such as the canine model for spine disorders, are the most favorable model for IVD diseases such as IVD degeneration and herniation, due to the same pathophysiological mechanisms of disease, diagnosis, prevention, and treatment [2,3]. Statistically, only 2.3 to 3.7% of all dogs admitted to veterinary hospitals suffer from intervertebral disc disease [4], unlike in humans where approximately 5% of all individuals are affected [5]. One of the IVD cases is intervertebral disc herniation (IVDH), which causes pain and compression of the spinal cord associated with sensory and motor deficits and even permanent loss of limb function [6]. The severity of the clinical signs of IVDH correlates with parenchymal damage of the spinal cord [7]. Clinical signs in the thoracolumbar part of IVDH include back pain, pelvic limb paresis, overfull bladder, involuntary urination, and sometimes fecal incontinence [8]. Clinical neurological examination is essential to identify the affected part of the vertebra and the location of the compressed nerve root. Diagnostic imaging techniques for detecting IVDH include magnetic resonance imaging (MRI) and computed tomography (CT), whereas radiographs are not reliable [4]. Nowadays, it is reported that deep learning artificial intelligence models can automatically detect, classify, and grade IVD from MRI scans, which is a fast and efficient strategy for lumbar IVD diagnostics [9]. Treatment of thoracolumbar disc herniation includes non-surgical options with analgesics and physical therapy or surgical methods such as mini-hemilaminectomy, which causes less damage to the adjacent soft tissue and creates a smaller fenestration in the vertebra [10]. Due to the multifactorial nature and the lack of effective biological/pharmacological therapy to prevent disc degeneration and potentially severe consequences of disease progression [11], there is a need to apply a systems approach to the diagnostics, progression, and treatment monitoring of IVHD in order to increase the knowledge of the biological processes behind the clinical signs and improve the effectiveness of the treatment of this condition.

Serum/plasma and blood are the predominant samples used in clinical practice for diagnostics procedures as they are easy to collect and a wide range of tests can be performed using these biofluids [12]. In addition, biomolecules in serum are a measurable indicator of physiological and/or pathological changes in an organism. They contain not only blood-specific molecules, but also tissue and organ leakage proteins, metabolites, RNAs, microelements, etc. [13]. Understanding the IVDH-related pathophysiological processes at the molecular level that can be observed in serum could enable the identification of effective markers for early disease detection and potentially effective preventive targeted therapeutic strategies.

Omics technologies have emerged as complementary strategies to genomics. In the attempt to understand diseases, the use of omics technologies, such as mass-spectrometry-based metabolomics and proteomics, combined with bioinformatics enables the comprehensive analysis of metabolites, peptides, and proteins in complex samples, providing detailed information about disease pathology at the molecular level [14,15]. The complementary strengths of proteomic and metabolomics technologies have already been described as a powerful tool for the investigation of multifactorial neurodegenerative conditions [16] as well as diseases related to disc degeneration [17] by analyzing tissues or biological fluids (e.g., serum or cerebrospinal fluid (CSF)). During intervertebral disk degeneration, many proteomic and metabolomic pathways are affected, particularly in relation to spinal cord compression, tissue injury, inflammation, and the neuropathic pain state [14,18]. Recognizing the importance of multi-omics data integration in terms of results quality and informativeness, in our previous CSF omics study, we developed and described the new integromics prediction model identifying the key CSF molecules (e.g., metabolites and proteins) related to canine IVDH. Those molecules are involved in amino acids and lipid metabolism, coagulation cascades, neuropathic pain, myelination, neurotransmission, and inflammatory response [14].

Apart from proteins and metabolites, trace elements are also involved in various physiological processes and play a regulatory, structural, and/or catalytic role. Zinc (Zn), the second most abundant trace element in an organism, is known to regulate immunological processes, is a cofactor of zinc-binding proteins involved in wound healing, and has an osteogenic effect by stimulating collagen matrix synthesis and bone mineralization [19,20]. Its deficiency is associated with oxidative stress and inflammation, particularly in neurological disorders [20]. Zn levels, among other trace elements, have been investigated in relation to intervertebral disc degeneration in humans [21]. However, to the best of the authors’ knowledge, the studies in dogs have focused on skin, neurological, hepatic, renal, ocular, and reproductive disorders [22].

The aim of this study was to use the integrative high-resolution MS-based serum untargeted metabolomics and proteomics and advanced bioinformatic tools to decipher the key serum molecular players in canine IVDH with diagnostic potential by monitoring dogs with acute paraplegia caused by severe IVD herniation and nerve compression. In addition, the concentration of circulating zinc in serum was determined as a micronutrient potentially related to the pathophysiology of IVDH (e.g., degenerative changes, tissue regeneration, immune response). Finally, an enzyme-linked immunosorbent assay was used to validate proteomic results by measuring the concentrations of zinc alpha 2-glycoprotein (ZAG), alpha-1-microglobulin/bikunin precursor (AMBP), and vitronectin (VTN) in the serum of healthy dogs and dogs with IVDH.

## 2. Materials and Methods

### 2.1. Study Design

**Figure 1:**
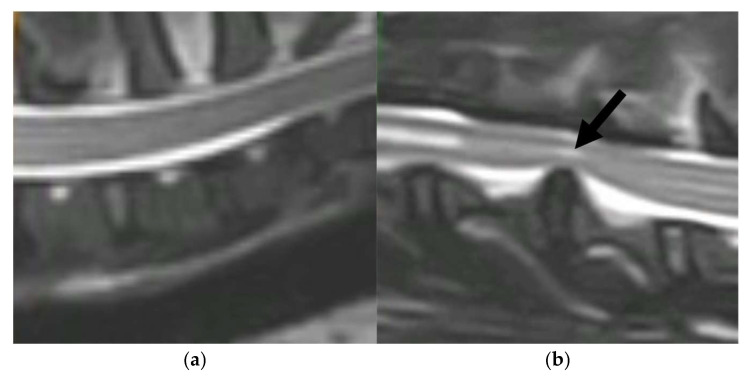
Computed tomography (CT) scans of (**a**) a healthy intervertebral disc in a dog and (**b**) a degraded intervertebral disc (marked by an arrow) that protrudes into the spinal canal and compresses the spinal cord and nerve root.

The clinical study involved 40 client-owned canine patients from our veterinary specialty clinic (Clinic for Surgery, Orthopedics and Ophthalmology, Faculty of Veterinary Medicine, University of Zagreb). Dogs were classified into two groups, healthy controls (*n* = 10) and the IVDH group (*n* = 13). The control group consisted of mixed breeds (average body weight 21 kg), age 2–7 years, while the IVDH group comprised mixed breeds and pure breeds (body weight 6–15 kg), age 6–12 years. The validation group consisted of *n* = 7 healthy dogs (mixed breeds, age 2–7 years) and *n* = 10 canine IVDH patients (mixed breeds and pure breeds, age 7–11). The dogs classified in the IVDH group were paraplegic with a preserved deep pain reflex, and all underwent a surgery procedure for decompression of the spinal cord. The IVDH group consisted of dogs with acute onset paraplegia caused by severe intervertebral disc herniation (severe nerve compression). All dogs in the IVDH group were presented to the surgery clinic within 24 h of the sudden acute onset of paraplegia. Samples were collected 24 to 48 h following the onset of paraplegia. All dogs included in this study were submitted to general physical examination, and complete blood count and blood biochemistry were performed. Dogs being diagnosed with any other pathological condition (chronic paraplegia, systemic and metabolic diseases) were excluded from this study. Intervertebral disc herniation was diagnosed in affected dogs in the thoracic–lumbar spine (T13/L1/L2) by clinical examination and confirmed by computerized tomography (CT) (Figure 1).

The owners signed a written consent for participation in the study and acceptance of the risk of anesthesia and surgical procedure. The research was approved by the Ethical Committee of the Faculty of Veterinary Medicine, University of Zagreb, Croatia (File No. 640-01/18-17/67; Case No. 251-61-21/333-18-01).

### 2.2. Serum Sample Collection

The skin was aseptically prepared prior to blood sampling from v. *Cephalica antebrachii*. Blood was taken into sterile SST serum separation tubes (DB Vacutainer^®^, Becton, Dickinson and Company, Franklin Lakes, NJ, USA). The serum was collected by centrifugation at 1200× *g* and aliquoted. Aliquots remaining from routine biochemical analysis were stored at −80 °C for further analysis.

### 2.3. Untargeted Metabolomic Analysis

LC-MS-based untargeted metabolomics analysis of canine serum samples (control *n* = 10 and IVDH *n* = 13) was performed as reported previously by our group [14,23]. In short, extraction solvent (chloroform/methanol/water; 1:3:1, *v*/*v*/*v*) was used as matrix blank. A pooled sample was prepared by mixing 10 µL of each serum sample. Metabolites were extracted from 25 µL of each sample (including pool and matrix blank) using extraction solvent by vortexing (2 h at 4 °C) and centrifugation (13,000× *g* for 5 min at 4 °C). The obtained extracts were stored at −80 °C until further analysis. For each sample, a volume of 10 μL of metabolite extract was injected onto a zwitterionic-polymeric hydrophilic interaction chromatography (ZIC-pHILIC) column (150 × 4.6 mm, particle size 5 μm; SeQuant), Merck KGaA, Darmstadt, Germany) using a Dionex UltiMate 3000 RSLC system (Thermo Fisher Scientific, Hemel Hempstead, UK) coupled to a Thermo Orbitrap Q Exactive Plus (Thermo Fisher Scientific). The column temperature was set at 25 °C, and the flow rate was 0.3 mL/min. The runtime was 26 min with the following LC gradient: 80% B (0 min), 5% B (15 min), 80% B (17 min), equilibration (26 min). Mobile phase A contained 20 mM ammonium carbonate aqueous solution, and B contained 100% acetonitrile. The mass spectrometer was operated with fast-changing positive and negative polarity with electrospray ionization (ESI) in the *m*/*z* range of 70–1050 and a resolution of 70,000. The source voltage was set at 3.8 kV, and the capillary temperature was 320 °C. Nitrogen was used as sheath gas (40 arbitrary units) and auxiliary gas (5 arbitrary units). For metabolite identification a mix of 148 authentic compounds, which was kindly donated by Dr. Richard Burchmore, Glasgow Polyomics, University of Glasgow, UK, was used.

Metabolomics raw data were further processed using the Polyomics integrated Metabolomics Pipeline (PiMP) standard workflow and default processing parameters as reported [14,24]. In short, collected LC-MS raw data were converted to ‘mzXML’ file format, centroided, and split into positive and negative polarities using the MSConvert tool [25]. Metabolite identification was performed in PiMP by matching the retention times and accurate masses of detected peaks with the authentic standards (Level 1), or metabolites were putatively annotated (based on accurate masses and MS/MS fragmentation data) using the Human Metabolome Database (HMDB), available at https://hmdb.ca/ (accessed on 14 May 2024) (Level 2). The metabolite functions were determined using the Kyoto Encyclopedia of Genes and Genomes (KEGG), available at https://www.genome.jp/kegg/ (accessed on 14 May 2024), integrated within PiMP. Level 3 metabolites were not used for further analysis; however, they are listed in Supplementary File S1 as annotated.

### 2.4. Serum Zinc Determination

The serum samples used herein were complementary to CSF samples from our previous study [14]. The concentration of zinc in the serum was determined following a diagnostic assay—the spectrophotometric direct colorimetric method without deproteinization (Sentinel CH., Milano, Italy) using Beckman Coulter analyzer DxC700 (Beckman Coulter, Brea, CA, USA). Differences between the control (*n* = 4) and IVDH (*n* = 6) groups for serum zinc concentrations were assessed by the Mann–Whitney U test within GraphPad Prism software (v.8.4.2.) (GraphPad Software Inc., San Diego, CA, USA).

### 2.5. High-Resolution Mass-Spectrometry-Based Proteomic Analysis

Tandem mass tag (TMT)-based quantitative proteomic analysis of canine serum samples was performed as reported by our group [23] using the serum samples matching CSF as described in our CSF study [14]. In short, total serum protein concentration was determined using BCA assay (Thermo Scientific, Rockford, IL, USA). For each sample, 35 µg of serum proteins was diluted using 0.1 M triethyl ammonium bicarbonate (TEAB, Thermo Scientific, Rockford, IL, USA), reduced (DTT; Sigma Aldrich, St. Louis, MO, USA), alkylated (IAA; Sigma Aldrich, St. Louis, MO, USA), and acetone-precipitated overnight. Protein pellets were dissolved in 50 µL of 0.1 M TEAB, digested overnight using trypsin (trypsin-to-protein ratio 1:35), and labeled using TMT tenplex reagents (Thermo Scientific, Rockford, IL, USA) according to the manufacturers’ instructions. TMT-labeled peptide samples were combined, aliquoted, dried, and stored at −80 °C until analyzed. LC-MS/MS analysis was performed using an Ultimate 3000 RSLCnano system (Dionex, Germering, Germany) coupled to a Q Exactive Plus mass spectrometer (Thermo Fisher Scientific, Bremen, Germany) as reported [26]. In short, labeled peptides were desalted on the trap column and separated on the analytical column (PepMap™ RSLC C18, 50 cm × 75 μm) over a 120 min linear gradient of 5–45% mobile phase B (0.1% formic acid in 80% ACN) at the flow rate of 300 nL/min. Mobile phase A consisted of LC-MS-grade water and 0.1% formic acid. A Nanospray Flex ion source (Thermo Fisher Scientific, Bremen, Germany) equipped with a 10 μm inner diameter SilicaTip emitter (New Objective, Littleton, MA, USA) was used for ionization. The Top8 DDA method in positive ion mode was applied. For full-scan MS acquisition, MS parameters were set as follows: a resolution of 70,000, a mass range of *m*/*z* 350.0–1800.0, a 120 ms injection time, an AGC target of 1 × 10^6^, and a ± 2.0 Da isolation window. The dynamic exclusion time was enabled for 30 s. For ion fragmentation, high-energy collisional dissociation (HCD) with step collision energy (29% and 35% NCE), a resolution of 17,500, and an AGC target of 2 × 10^5^ were used.

Protein identification and reporter-ion-based relative quantification were performed in Proteome Discoverer (version 2.3., Thermo Fisher Scientific) using a SEQUEST database search (*Canis lupus familiaris* FASTA, downloaded from NCBI database 3 May 2022, 172,099 entries). Search parameters were set as follows: two trypsin missed cleavage sites; precursor and fragment mass tolerances of 10 ppm and 0.02 Da, respectively; fixed modification: carbamidomethyl (C); dynamic modifications: TMT sixplex (K, peptide N-terminus) and oxidation (M). The false discovery rate (FDR) for peptide identification was set at 1%. Proteins containing at least two unique peptides and 1% FDR were reported as identified and used for subsequent statistical and bioinformatic analyses.

### 2.6. Analytical Validation of Proteomic Results

Canine-specific enzyme-linked immunosorbent assays (ELISAs) were used for the determination of zinc alpha 2-glycoprotein (ZAG) (BlueGene Biotech, Shanghai, China), alpha-1-microglobulin/bikunin precursor (AMBP) (BT Lab, Jiangxing, China), and vitronectin (VTN) (BT Lab, Jiangxing, China) concentrations in validation group of samples containing healthy (*n* = 7) and IVDH (*n* = 10) canine serum according to manufacturer’s instructions. Differences between the control and IVDH groups were assessed by the Mann–Whitney U test within GraphPad Prism software (v8.4.2.) (GraphPad Software Inc., San Diego, CA, USA).

### 2.7. Statistical and Bioinformatic Analyses

***Metabolomics:*** Metabolomics data were analyzed and visualized according to untargeted metabolomics workflow guidelines [27]. Raw data were submitted to the PiMP for metabolite annotation, statistical analysis, and metabolic pathway analysis using a standard PiMP workflow [16]. One group-wise comparison was undertaken to identify differences between the IVDH and control groups. Peaks with a Benjamini–Hochberg-adjusted *p*-value less than 0.05 were considered significant. However, only identified metabolites (by matching to a standard compound and/or presumptively annotated by database search using fragmentation spectra, e.g., Level 1 and Level 2 according to MSI) were used for subsequent bioinformatic analysis. Further visualization (heatmap) of PiMP-exported data was performed using R v4.3.2 and the pheatmap package v1.012. Volcano plots were created using the ggplot2 package v3.5.2. Bar charts for statistically differentially abundant metabolites were directly exported from PiMP. Partial least-squares discriminant analysis (PLS-DA) was performed in MetaboAnalyst (v6.0.) [28]. Peak intensity data were exported from PiMP using the default MetaboAnalyst export function. The data were processed following MetaboAnalyst workflow. Peaks having more than 50% missing values were filtered out, and KNN as the missing value imputation method, logarithm transformation, and Pareto scaling were applied as recommended for untargeted metabolomics data analysis [27].

***Metabolite Interactions and Pathway Analysis*:** Metabolite–metabolite interaction and pathway analyses were performed using the Pathway Analysis tool implemented in MetaboAnalyst. The pipelines used were based on the human KEGG metabolic pathways library. The over-representation data were obtained by hypergeometric tests. The topology measurement was performed using relative-betweenness centrality (the number of shortest paths going through the node focusing on global network topology).

***Proteomics:*** Proteins having more than 50% missing values were excluded from the subsequent statistical analysis. Statistics were performed using R (v3.2.2) with a custom analysis script. Sample outliers were detected using the R package outliers v0.14 [29] for each group for each protein using Dixon’s test, and significant outliers (*p* < 0.05) were removed. The fold change (FC) in protein abundance between two groups was determined by the Mann–Whitney U test and expressed on a log2 scale. The design of PCA and volcano plots was performed using the R package ggplot2 v3.5.2 [30]. For bioinformatic analysis, proteins’ GI accession numbers obtained by database search were converted into official gene symbols (DAVID conversion tool (https://david.ncifcrf.gov/conversion.jsp, accessed on 11 February 2025) and Uni-ProtKB ID mapping (https://www.uniprot.org/uploadlists/, accessed on 11 February 2025). Protein–protein interaction and REACTOME pathway analyses were performed using STRINGdb v11.0 (https://string-db.org/, accessed on 11 February 2025) [31], with the selection of *Canis lupus familiaris* default settings with the exception of no more than 5 interactors showing in the 1st shell. Networks of the relationship between the REACTOME pathway and proteins with significantly differential abundances between groups were designed using Cytoscape v.3.7.1. (https://cytoscape.org/, accessed on 12 February 2025) [32]. Additionally, Gene Ontology (GO biological process, GO molecular function, GO cellular component) enrichment analysis was performed using clusterProfiler v4.10.0. First, biomaRt v2.63.0 [33] with the Ensembl database was used to convert gene names to Entrez ID and then to human orthologs. GO over-representation tests were performed separately for up- and downregulated DAPs, and the results were filtered based on FDR-adjusted *p*-value < 0.05. Redundant GO terms were removed by applying the semantic similarity method implemented within the function simplify, using the similarity cut-off of 0.5.

## 3. Results

### 3.1. Untargeted Serum Metabolomics of Canine IVDH Patients

**Figure 2:**
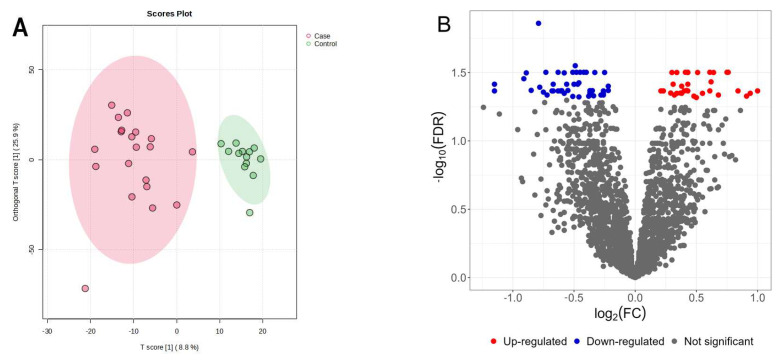

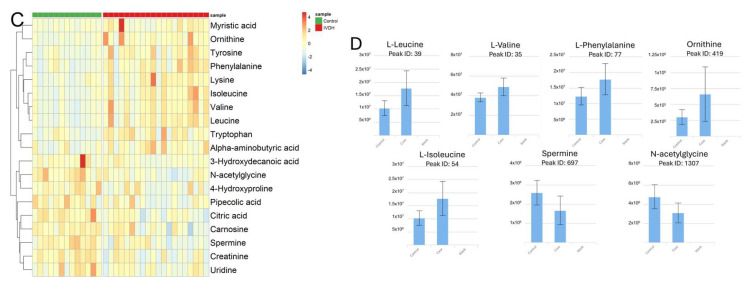
(**A**) Partial least-squares discriminant analysis (PLS-DA) score plot of the identified metabolites showing that dogs with IVDH (in red) differed from controls (in green), (**B**) the volcano plot of differentially expressed metabolites between dogs with IVDH and healthy dogs, (**C**) the heatmap of differentially abundant metabolites in control and IVDH samples, and (**D**) the intensity comparison of significantly deregulated metabolites (FDR-adjusted *p* < 0.05) in the serum of dogs with IVDH and healthy dogs (generated by PiMP software).

Untargeted metabolomic analysis using the HILIC-LC-MS platform resulted in the detection of 2341 peaks (features). After PiMP processing, features were identified and/or annotated based on mass (Level 2) and mass/retention time matched to known standards (Level 1). A total of 44 unique compounds were matched to a known standard (Level 1), and 484 metabolites were putatively annotated by HMDB database search using the Fragment Annotation Kit integrated within PiMP (Level 2). Metabolite identification results are available in Supplementary File S1. A partial least-squares discriminant analysis (PLS-DA) score plot (Figure 1a) shows a clear separation of serum metabolites originating from healthy (*n* = 10) and IVDH (*n* = 13) dogs. The volcano plot showing the differentially abundant metabolites (DAMs) and the intensity comparisons of DAMs (downloaded from PiMP) for each of the seven significantly altered metabolites are depicted in Figure 2B,C.

Table 1 shows the statistically significant identified and annotated metabolites together with their associated log2 fold changes. Metabolites with an adjusted *p*-value of less than 0.05 were considered significant, while those with *p*-values less than 0.05 were considered suggestively changed. Higher levels of ornithine, isoleucine, leucine, phenylalanine, and valine and lower levels of N-acetyl glycine and spermine were found to be statistically significant in the serum of dogs with IVDH. In addition, higher concentrations of lysine, tyrosine, tryptophan, alpha-aminobutyric acid, and myristic acid and lower concentrations of citric acid, uridine, creatinine, 3-hydroxydecanoic acid, carnosine, pipecolic acid, and 4-hydroxyproline were also detected. However, after FDR correction, the changes were not statistically significant compared to metabolites from the control group. Although these metabolites are not significantly altered, the concentration shift trend provides additional information and confirms the altered biological pathways observed in the serum of dogs with IVDH.

The serum metabolites listed in Table 1 were further analyzed using a compound list to create a metabolite–metabolite interaction network in MetaboAnalyst. Functions of the upregulated and downregulated metabolites were further explored in KEGG to gain a detailed insight into the affected metabolic pathways related to IVDH. The results obtained are shown in Table 2 (and are also available in Supplementary File S1). The metabolite interaction network revealed that the metabolites whose concentration was increased in IVDH were related to lipoic acid metabolism (*p* = 1.47 × 10^−9^), nitrogen metabolism (*p* = 9.43 × 10^−6^), and steroid biosynthesis (*p* = 2.06 × 10^−4^), based on their function. The metabolites that occur in lower concentration in dogs with IVDH are mostly involved in fatty acid biosynthesis (*p* = 4.63 × 10^−3^).

**Figure 3:**
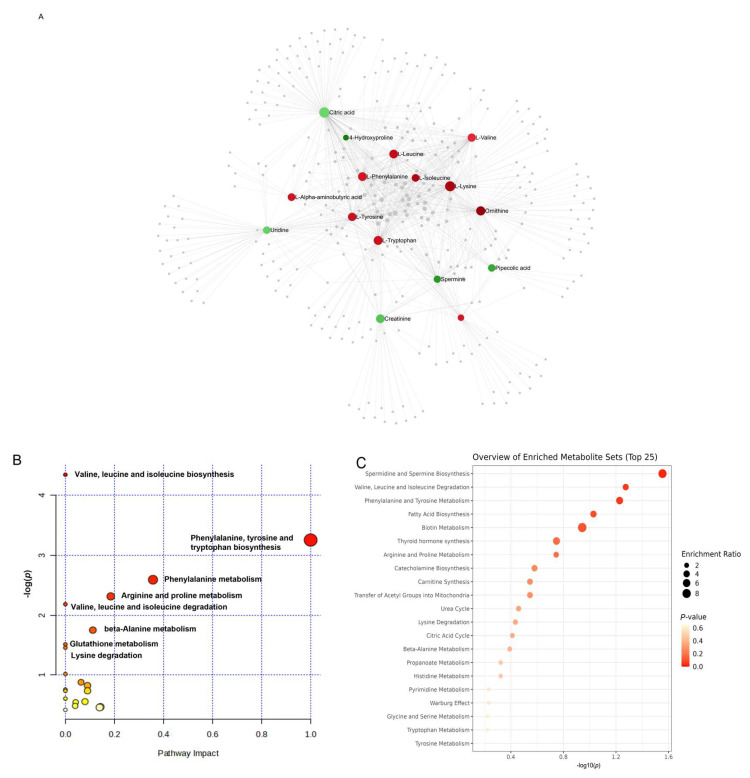
(**A**) Metabolite–metabolite interaction of upregulated (red dots) and downregulated (green dots) differentially abundant metabolites in the serum of IVDH vs. healthy dogs generated in MetaboAnalyst using metabolites listed in Table 1 as input data. Relevant pathways (without (**B**) and with enrichment (**C**)) altered in IVDH obtained based on the differentially abundant metabolites. The size of the node, pathway impact, and enrichment ratios were generated by topology analysis. The color of each node depicts the statistical significance in terms of log10 (*p*-value).

Furthermore, enrichment and pathway analyses of deregulated metabolites were also performed in MetaboAnalyst, providing data on IVDH-affected metabolic pathways. KEGG pathway analysis of serum metabolites altered in IVDH (Figure 3) showed that valine, leucine, and isoleucine biosynthesis; phenylalanine, tyrosine, and tryptophan biosynthesis; spermine and spermidine biosynthesis; and fatty acid biosynthesis are the most significant metabolic pathways altered in IVDH that could be observed in serum. Of those, phenylalanine, tyrosine, and tryptophan biosynthesis was shown to have the highest impact.

### 3.2. Serum Zinc Levels

**Figure 4:**
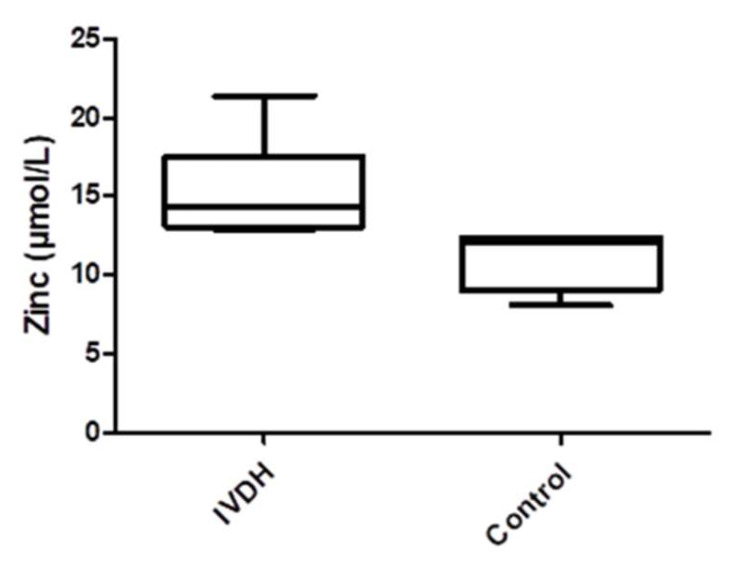
Median serum concentrations of serum Zn determined by colorimetric method in dogs with IVDH compared to healthy controls.

Serum zinc concentrations determined by the spectrophotometric method were significantly higher (*p* = 0.0095) in the IVDH group (median, interquartile range: 14.35 µmol/L, 13.05–17.58 µmol/L) compared to controls (12.10 µmol/L, 9.01–12.40 µmol/L), as depicted in Figure 4.

### 3.3. Proteomic Analysis

**Figure 5:**
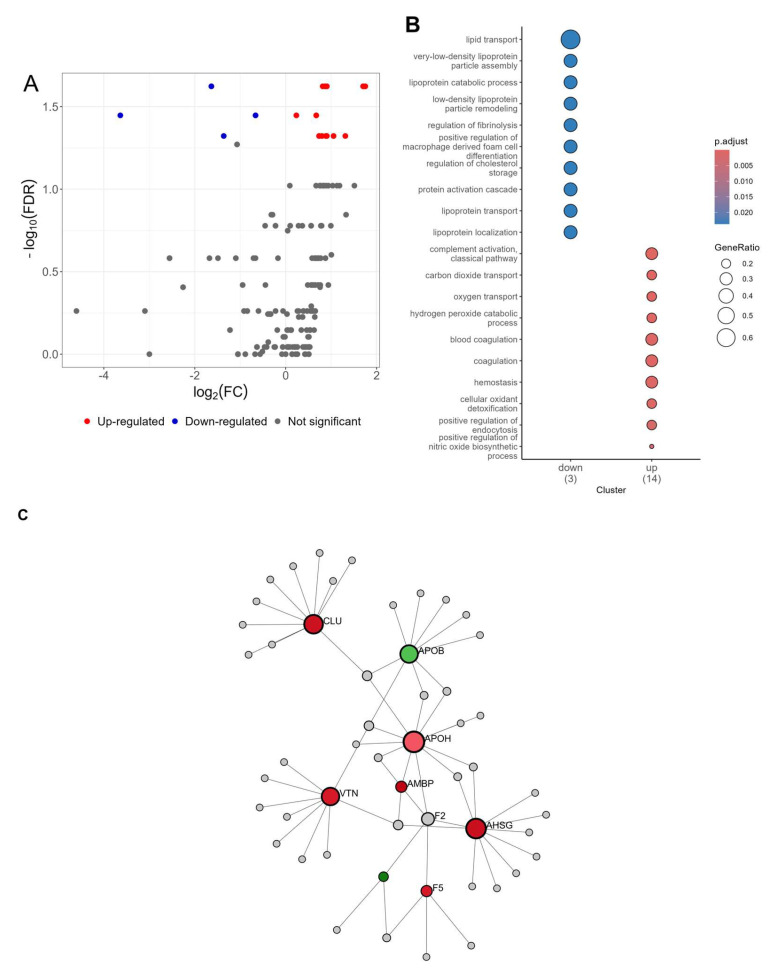
(**A**) Volcano plot of differentially expressed metabolites between dogs with IVDH and healthy dogs, (**B**) Gene Ontology (biological process, BP) of differentially abundant serum proteins due to IVDH (details provided within Supplementary File S2), and (**C**) protein interaction network generated using differentially abundant proteins (red nodes—up; green nodes–down) generated in Network Analyst based on STRING database.

Proteomic analysis of canine serum was performed using data-dependent analysis (DDA) by applying a TMT-label-based proteomic approach. This approach enabled us to identify 323 serum proteins (two unique peptides, 1% FDR). After applying FDR correction, statistical analysis revealed 27 significantly differentially abundant proteins (DAPs) in IVDH group compared to healthy dogs, 21 of which were higher and 6 lower in abundance in IVDH, as shown in Table 3. The volcano plot and heatmap of DAPs are depicted in Figure 5. Detailed information on identified proteins is provided in Supplementary File S2.

Gene Ontology over-representation analysis of DAPs revealed their involvement in lipid transport and metabolism for downregulated DAPs, while coagulation, oxygen transport, and complement activation were enriched for upregulated DAPs. The same was observed using the Reactome Pathways database, where the upregulated serum proteins in canine IVDH determined herein were mostly involved in platelet degranulation, activation, signaling, and aggregation; fibrin cloth formation; and the scavenging of heme from plasma, while the downregulated proteins were involved in lipid metabolism (Table 4).

### 3.4. Metabolomics and Proteomics Data Integration

**Figure 6:**
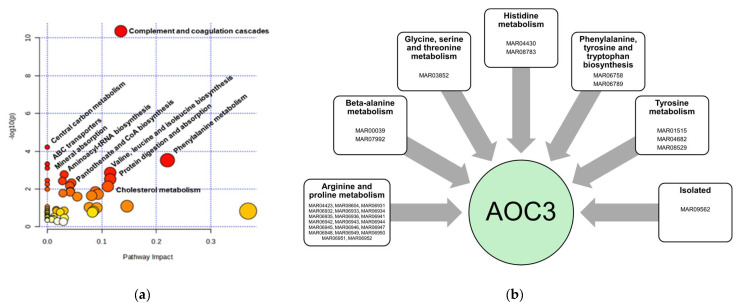
(**a**) Joint pathways in canine serum altered in IVDH obtained based on the differentially abundant proteins and metabolites. The size of the node, pathway impact, and enrichment ratios were generated by topology analysis in MetaboAnalyst. The color of each node depicts the statistical significance in terms of log10(*p*-value). (**b**) A protein upregulated in IVDH, amine oxidase copper containing 3 (AOC3), is involved in multiple metabolomic pathways. By accessing the Supplementary File S3 (or Metabolic Atlas reaction IDs within the pathway), the reaction equation, as well as the related genes and cellular compartment involved, can be reached (https://metabolicatlas.org/explore/Rat-GEM/gem-browser/gene/Aoc3, accessed on 28 April 2025).

Joint pathway analysis of significantly deregulated genes and metabolites (Figure 6) revealed that the complement and coagulation cascade (*p* = 4.407 × 10^−11^); central carbon metabolism in cancer (*p* = 6.060 × 10^−5^); phenylalanine metabolism (*p* = 2.993 × 10^−4^); ABC transporters (*p* = 4.7342 × 10^−4^); mineral absorption (*p* = 7.7898 × 10^−4^); and valine, leucine, and isoleucine biosynthesis (*p* = 0.00137), among other pathways, are altered in IVDH.

Among the deregulated proteins, according to Metabolic Atlas database (Rat-GEM, including 13,026 reactions, 8371 metabolites and 8218 genes) [34], only amine oxidase copper containing 3 (AOC3) was found to be directly involved in metabolic pathways, mostly related to arginine and proline metabolism, especially in metabolism of polyamines, e.g., spermidine and spermine oxidation in which H_2_O_2_ and NH_3_ are released. Other metabolic pathways involving AOC3, together with the reaction details, can be found in Figure 6 and Supplementary File S3.

### 3.5. Analytical Validation of Proteomic Results by ELISA

**Figure 7:**
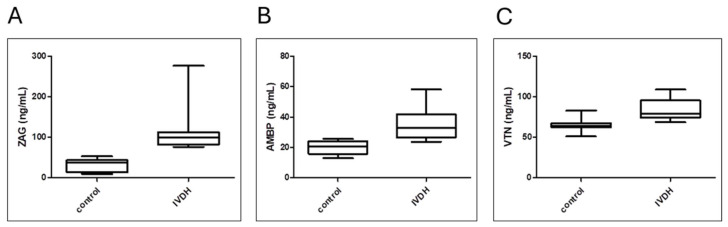
Median serum concentrations of (**A**) zinc alpha 2-glycoprotein (ZAG), (**B**) alpha-1-microglobulin/bikunin precursor (AMBP), and (**C**) vitronectin (VTN) determined by ELISA in dogs with IVDH compared to healthy controls. *p*-values were determined as follows: *p* = 0.0001, 0.0004, and 0.0031, respectively.

The validation results obtained by an immunoaffinity approach (ELISA) aligned with the proteomics findings, showing (Figure 7) significantly higher concentrations of ZAG, AMBP, and VTN in the IVDH group compared to controls, as reported in Table 5.

## 4. Discussion

Despite the relatively low prevalence of IVDH in dogs (2%), the focus on canine IVDH has turned to various research interests in recent years, including animal welfare and dog model animals for degenerative diseases in humans and innovative treatment strategies [35]. The severity of disease in dogs influences the prognosis in terms of treatment (drug or surgical therapy), recovery after surgery, or euthanasia. In some countries, X-ray screening has been introduced to reduce the incidence of IVDH by excluding dogs at high risk of developing IVD-related diseases from use for breeding purposes. Although this is a positive step for animal welfare, this approach is not entirely accurate or effective [36], demanding reliable targeted molecules (gene, protein, metabolite) for accurate screening.

Due to IVDH’s multifactorial nature, its pathophysiological mechanisms are still poorly understood. Furthermore, from a clinical perspective, there is an urgent need for a non-invasive biomarker that can predict clinical outcome [37]. Due to the limited instrumentation and ideally matched canine patients available to veterinary clinicians, as well as ethical constraints (particularly in obtaining clinical samples from healthy dogs), clinical studies of IVDH have mostly focused on specific molecules in serum, which can be monitored using general biochemistry (CRP) [38] or ELISA tests (serum levels of the phosphorylated form of the high-molecular-weight neurofilament subunit NF-H, pNF-H) [39], hypothesizing that the change in their concentration is associated with the pathological processes (e.g., spinal cord injury). The introduction of high-throughput methodology and the systems approach in the field of veterinary medicine has increased the number of omics studies and facilitated the understanding of molecular processes at the molecular level in relation to pathophysiology [40,41]. To the best of the authors’ knowledge, omics studies involving canine IVDH are very scarce. Apart from proteomics and metabolomics studies related to IVD [42], there are a few recent studies focused on investigating the serum metabolome of IVDH in humans [43]. In addition, several studies addressing proteomics and metabolomics of serum and CSF have been published in relation to IVD extrusion-induced spinal cord injury and neurological pain. With this in mind, in our previous study, we performed an integrated proteomic and metabolomic analysis of canine CSF, using machine learning to pinpoint key molecules of IVDH. In addition, this study aimed to identify metabolomic and proteomic alterations to explore the key molecules and metabolic and biological pathways involved in canine IVDH that could be observed in sera of dogs with acute paraplegia due to severe IVD herniation and nerve compression.

### 4.1. Metabolic Pathways Altered in IVDH

Based on the metabolite–metabolite interaction of nineteen metabolites that are altered in the serum of dogs with IVDH, we found that lipoic acid metabolism, especially the part related to branched-chain amino acid (BCAA) metabolism, is most altered in IVDH. BCAAs can be catabolized or delivered to muscle cells and incorporated into proteins [44]. The role of lipoic acid metabolism is to provide enzyme-linked cofactors for 2-ketoacid dehydrogenases, among which is branched-chain ketoacid dehydrogenase (BCKDH), and is essential for the regulation of protein and carbohydrate metabolism, as well as energy production [45]. To support these metabolic changes, our study also found an increase in lipoic acid in the serum of IVDH dogs, although not this increase was not significant (Peak ID 133; log2FC = 0.68, *p* = 0.051, Supplementary File S1). Lipoic acid is an antioxidant involved in the regeneration of endogenous antioxidants, ROS scavenging, and metal chelation [45]. Herein we found that the concentration of valine, leucine, and isoleucine is significantly increased in the serum of IVDH dogs compared to healthy dogs. BCAAs play an important physiological role in promoting protein synthesis in muscle under conditions of negative energy balance or greater physical exertion through increased fatty acid oxidation [46]. In muscle, transamination to 2-oxo (or -keto) acids occurs, followed by oxidative decarboxylation resulting in the formation of coenzyme A (CoA) derivatives by BCKDH. BCAAs have been reported to play an important role in nucleoside metabolism, as well as in nitrogen metabolism, as they are nitrogen donors in the process of neurotransmitter synthesis and the glutamate/glutamine cycle [47]. The intermediates of BCAA degradation are also involved in gluconeogenesis, lipogenesis, and the cholesterol synthesis pathway [48]. Dysregulation of BCCA metabolism has been reported to contribute to the development of neurodegenerative diseases such as Parkinson’s and Alzheimer’s diseases and amyotrophic lateral sclerosis by inducing oxidative stress, inflammation, and hyperexcitability [49]. Our study also found uridine concentration was lower in the serum of dogs with IVDH that had an elevated serum BCAA concentration. Uridine is a pyrimidine nucleotide present in blood and CSF that has an anti-inflammatory effect, reduces cytotoxicity, and improves the neurophysiological functions of peripheral nerves. It is also associated with glucose homeostasis and lipid metabolism [36,38]. Elsewhere, oral intake of BCAAs has been reported to reduce plasma uridine concentration without altering the concentration of glucagon, insulin, or purine bases (xanthine, hypoxanthine, or uric acid). However, the exact mechanism has not been clarified [36]. Among the BCAAs, leucine was also found to be significantly elevated in the serum of dogs with IVDH. Leucine is crucial for muscle growth activating mammalian target of rapamycin (mTOR), a stimulator of protein synthesis and a key protein involved in inflammation-dependent muscle regeneration [50]. In rats, leucine-enriched amino acids have been shown to improve recovery from muscle damage by modulating inflammation [51].

**Figure 8:**
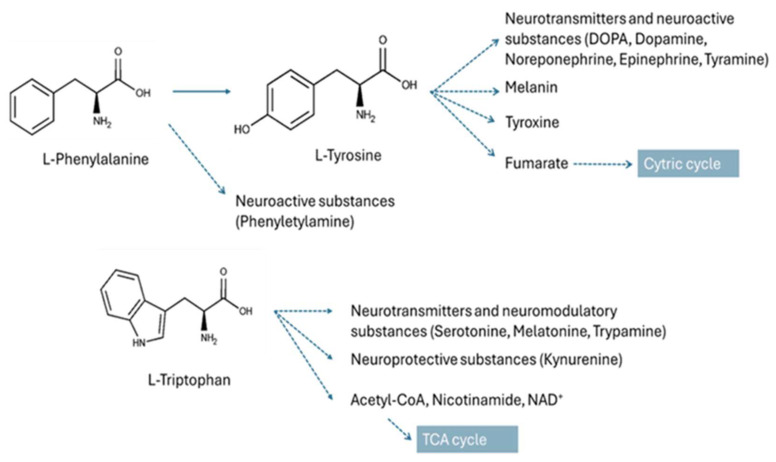
Phenylalanine, tyrosine, and tryptophan metabolism provides a variety of metabolites for key physiological processes.

Pathway analysis of the deregulated metabolites revealed phenylalanine, tyrosine, and tryptophan biosynthesis being a pathway altered in IVDH with the highest impact (Figure 7). These neutral aromatic amino acids are all elevated in the serum of IVDH canine patients. The aromatic amino acids, besides being involved in protein synthesis, are also starting points in catabolism, providing secondary metabolites that have key roles in organisms [52]. Phe can be converted to Tyr, and Trp and Tyr are precursors for the synthesis of neurotransmitters and neuroactive substances, as shown in Figure 8 [53,54]. They have been strongly associated with severe pathological manifestations such as neuropathological conditions (Trp), seizures and mental disorders (Phe, Tyr), albinism, and decreased immune defense (Tyr) [52]. It has been reported that elevated levels of Phe, Tyr, and Trp are correlated with a neuropathic pain-relieving effect in rats [55].

**Figure 9:**
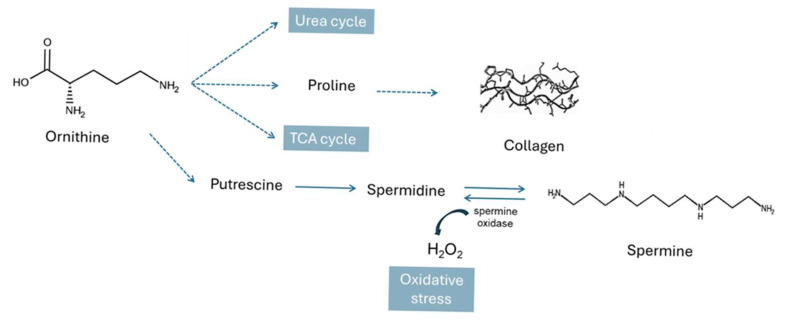
The involvement of ornithine in various metabolic pathways including collagen and polyamine synthesis.

In addition, spermidine and spermine biosynthesis (Figure 8) is also found to be altered in dogs with IVDH in our study. Spermine is an endogenous cationic polyamine that is involved in a variety of physiological processes. Under physiological conditions, it binds negatively charged nucleic acids and phospholipids. It is not only associated with the regulation of gene expression and inhibition of DNA from singlet oxygen damage [56], but it also acts directly as a radical scavenger [57]. Spermine is also involved in the transport of metal ions (e.g., potassium and calcium) by altering the functions of ion channels and receptors that directly control membrane potential. However, an oxidation product of spermine, hydrogen peroxide, can trigger cell damage and programmed cell death [58,59]. Altered polyamine homeostasis (e.g., putrescine, spermidine, and spermine) has been observed in neurodegenerative diseases and traumatic brain injury [60]. It has been reported that increased spermine concentration can induce neuronal depolarization and cytoplasmic calcium ion overload, leading to mitochondria-related injury and neuronal damage [60]. In our study spermine was found to be significantly lower in the serum of IVDH dogs. This can be explained by the presence of oxidative stress caused by the increased production of the reactive oxygen species (ROS) hydrogen peroxide (H_2_O_2_) associated with the catalytic activity of spermine oxidase (SMOX) [61]. SMOX oxidizes spermine to spermidine, which results in the formation of hydrogen peroxide and aldehyde 3-aminopropanal, which can potentially cause various pathologies including cellular or neuronal damage with a decrease in spermine and an increase in spermidine concentration. This mechanism has already been observed in the degeneration of retinal neurons in rats [62]. The authors suggested that SMOX expression could be increased during inflammation or under stress conditions caused by DNA damage. In our case, this needs to be further confirmed by monitoring spermidine concentration; however, spermidine was not identified herein. In this context of polyamine synthesis, ornithine (which is converted to putrescine and then to spermidine and spermine [63]), was found to be significantly elevated in the serum of IVDH dogs. Ornithine (Figure 9) plays an important role in many key processes in the mitochondria, e.g., as an intermediate in the urea cycle, and in proline synthesis. An increase in circulating serum ornithine concentration has been associated with inflammation and musculoskeletal pain [64,65].

Proline is essential for the biosynthesis of proline-containing proteins, as well as collagen, which maintains the structural support of bones, blood vessels, and skin. Apart from maintaining tissue integrity, collagen also plays an important role in wound healing [66]. To achieve its structural stability, collagen contains hydroxylated amino acids (mainly at proline residues) by increasing the density of hydrogen bonding groups and strengthening the electrostatic interactions between triple-helix monomers [67]. Although collagen degradation leads to an increased concentration of hydroxyproline in CSF, as reported in our previous study, the free circulating serum hydroxyproline was found to be lower in the serum of dogs with IVDH. Proline and its hydroxylated form hydroxyproline have been reported to regulate collagen biosynthesis by increasing the amount of TGF β1 receptors. In our case, it can be hypothesized that collagen synthesis in dogs was reduced by hydroxyproline deficiency. A similar finding related to low serum hydroxyproline was reported in the study of rheumatoid arthritis related to joint damage in humans, describing a decline in free and a rise in protein-bound hydroxyproline in serum.

In contrast to the upregulated mechanisms, fatty acid biosynthesis was found to be downregulated in the serum of dogs with IVDH, mainly due to lower levels of 3-hydroxydecanoic acid. 3-Hydroxydecanoic acid is a medium-chain fatty acid and an important mediator of inflammation. It has been reported that 3-hydroxydecanoic acid is a ligand activating proinflammatory G protein-coupled receptor 84 (GPR84) [68]. As in our study, in which the concentration of 3-hydroxydecanoic acid was found to be lower in the serum of IVDH, the downregulation of this oxidized fatty acid was also found in asymptomatic COVID-19 patients. It has also been shown that, along with several other fatty acids, 3-hydroxydecanoic acid significantly reduced H_2_O_2_ production [69]. However, the accumulation of 3-hydroxy fatty acids in cells or neurons can induce oxidative stress, mitochondrial dysfunction, and lipotoxicity [70]. In IVDH, oxidative stress is present, causing ROS production, and this could affect levels of 3-hydroxydecanoic acid. However, it would also be interesting to monitor levels of accumulated fatty acid in damaged tissue to obtain a more accurate conclusion.

Among the pathways, lysine metabolism was also found to be altered in IVDH. Lysine is a proteinogenic amino acid that is responsible for building muscle and lysine-rich brain proteins, enzymes, and antibodies and has a plethora of other functions, including calcium absorption, hormone production, and the regulation of inflammation [71]. In an osteoarthritis chondrocyte model, Lys improved the hypertrophic transformation of chondrocytes by modulating matrix proteins and inflammatory cytokines by restoring collagen (type II collagen/type I collagen ratio) and aggrecan expression levels [72]. In addition, it was reported that in combination with arginine, lysine increased collagen production by altering the activity of bone-forming cells through calcium ion absorption [73]. The authors investigated the neurotropic effects of lysine, related to pain-induced behavior in rats, through its interaction with neurotransmitters that play an important role in the formation of agonistic behavior [71]. Although lysine levels were found to be elevated in the serum of IVDH dogs, the concentration of pipecolic acid, a metabolic intermediate of lysine degradation, was found to be lower, indicating that lysine degradation in peroxisome was not increased. However, the degradation of pipecolate by pipecolic acid and sarcosine oxidase generates H_2_O_2_. The overproduction of ROS can lead to protein, lipid, and DNA oxidative damage. The positive correlation of the elevated levels of pipecolic acid in serum with pyridoxine-dependent seizures was reported, revealing its importance in neuromodulation, e.g., in the presynaptic mechanism of GABA release [74].

### 4.2. Serum Proteome Changes in Canine IVDH

Proteomic results revealed that lipid transport and metabolism were downregulated while coagulation, complement cascade, and oxygen transport were upregulated in dogs with IVDH in our study. Out of 27 DAPs, ZAG, AMBP, and VTN were selected for validation based on their disease-related biological significance and test availability. Zinc-α2-glycoprotein (ZAG) is a secretory protein involved in many molecular pathways stimulating lipolysis and plays a role in immunomodulation, cell adhesion, and tumor proliferation. Its overexpression inhibits the mTOR pathway, which regulates the growth, metabolism, and cell–cell interactions of neurons and glia. ZAG is an anti-inflammatory cytokine that suppresses seizure via inhibiting neuroinflammation, with a possible use for epilepsy [75]. Elevated serum levels of ZAG were obtained herein by both proteomics and ELISA. The ZAG protein contains high-affinity binding sites for zinc (Zn). Circulating Zn concentrations in serum are crucial for maintaining ZAG activity and facilitate its binding to fatty acids [76]. In addition, Zn plays an important role in immune response, wound healing, pain signaling, and neuropathic pain [77]. Zn has also been found to promote neuronal recovery by reducing ROS levels in spinal cord tissue after spinal cord injury, while increasing superoxide dismutase activity and glutathione peroxidase production [78]. Here, serum Zn levels were determined. The concentration of Zn in healthy dogs was consistent with reported data for healthy dogs, in which the authors found no effect of age, breed, lifestyle, weight, or body condition score on serum zinc concentration [79]. Although the Zn concentration is within the physiological range (4.9–19.7 μmol/L), IVDH canine patients have significantly higher circulating Zn levels than healthy dogs.

AMBP, which is found to be elevated in the serum of dogs with IVDH, plays a role in the regulation of inflammatory processes. It is a precursor of the heme-binding protein α-1-microglobulin (A1M) and bikunin, which inhibits the production of proinflammatory cytokines and the contraction of vascular smooth muscle by blocking cellular calcium uptake. The increased concentration of AMBP in CSF was positively correlated with cerebral hemorrhage, and it was also elevated in the serum and CSF of patients with pre-eclampsia having neurological symptoms [80]. The authors’ hypothesis was that an increased level of free hemoglobin damages the brain–blood barrier by inducing oxidative stress. The increase in heme-scavenging protein concentration in dogs with IVDH could therefore be related to oxidative stress reduction. In support of this, hemoglobin levels were also found to be elevated in the serum of dogs with IVDH herein.

Vitronectin (VTN) is a glycoprotein present in the extracellular matrix and in blood. It has numerous functions, including regulating plasminogen activation; promoting cell adhesion, spreading, and migration; and participating in fibrinolysis and immune defense. It plays a key role in tissue repair and wound healing and promotes oligodendrocyte differentiation during the neurogenesis of human embryonic stem cells [81]. VTN was reported to play an essential role in regulating neuronal activities and functions. Keasey et al. showed that VTN leaks to the brain after vascular injury and activates leukemia inhibitory factor (LIF) and proinflammatory interleukin 6 (IL-6), triggering inflammation and subsequent repair [82]. VTN was found to be elevated in the serum of dogs with IVDH, which was confirmed by ELISA.

Joint pathways analysis based on deregulated proteins and metabolites revealed that the complement and coagulation cascade was the pathway that was most altered in IVDH. According to GO, the proteins involved in the regulation of the complement and coagulation cascade are C6, C1R, C1S, and CLU (all upregulated in the serum of IVDH canine patients). The proteins C1s and C1r form the calcium-dependent complex C1, which is the first component of the classical pathway of the complement system. The role of the activated complement in disc degeneration has been reported, and its mechanisms have been reviewed in detail [83]. Tissue injury triggers necrotic cell death, blood clotting, platelet activation, and an immunological response [84]. Complement system activation may further contribute to chronic inflammatory processes, fibrosis, angiogenesis, and neurogenesis. KLKB1 and SERPINA5 are involved in the formation of fibrin cloth and were found to be downregulated in the serum of dogs with IVDH. SERPINA5 acts as a procoagulant and proinflammatory factor by inhibiting the anticoagulant activated protein C factor as well as the generation of activated protein C factor by the thrombin/thrombomodulin complex [85]. The lower level of SERPINA5 could be related to activation of the anticoagulant protein C pathway, which was already reported in acute vascular damage. In addition to the activation of the intrinsic coagulation cascade through the activation of factor XII (FXII) and thrombus formation, serum prekallikrein KLKB1 plays a key role in vasodilation, vessel permeability, and inflammation [86,87]. KLKB1 is cleaved by activated factor XII (FXIIa) and serves as a precursor of plasma protease kallikrein, which cleaves kininogen into bradykinin. Bradykinin is a vasoactive peptide that causes vascular leakage and angioedema by binding to its receptor on endothelial cells. It has been shown that prekallikrein deficiency reduces thrombus formation in venous and arterial thrombosis mouse models [88]. Serum prekallikrein has also been associated with systemic and brain inflammatory processes [89].

In addition to the deregulated proteins mentioned above, amine oxidase copper containing 3 (AOC3), which was found to be upregulated, is also worth mentioning. It is not only involved in multiple metabolic pathways that preferentially metabolize polyamines (spermine and spermidine), but it also produces aldehyde, amine, and H_2_O_2_, which can induce oxidative stress and stress-activated pathways leading to cell death by apoptosis or necrosis [90]. It has been shown that AOC3 inhibition reduces oxidative stress and pain. In addition, the oxidase activity of AOC3 was positively correlated with the development of fibrosis in a bleomycin-induced pulmonary fibrosis mouse model [91].

### 4.3. Compilation of Serum and CSF Omics Results Reveals a Cascade of Events Related to Canine IVDH

To gain comprehensive insight into molecular processes related to IVDH that can be observed in the body fluids serum and CSF, we combined the results presented here with the proteomic and metabolomic results from our previous CSF omics study, taking into account that multiple serum and CSF samples (both control and IVDH samples) were complementary.

The complex cascade of events associated with intervertebral disc herniation, spinal cord injury, and neuropathic pain, as well as various treatment strategies, has been reviewed elsewhere [92]. In short, from the pathophysiological point of view, trauma (disc extrusion) causes the mechanical disruption of the blood–brain barrier (elevated concentration of hemoglobin in serum) and injury of the spinal cord, causing nervous tissue and blood vessel damage as well as inflammation leading to neuropathic pain. These processes lead to a series of vascular and biochemical changes related to the immune response, oxidative stress caused by reactive oxygen species (ROS) production, and ionic imbalance, as well as myelin repair and muscle regeneration [92], which is explained in detail here and in our previous multi-omics canine IVDH cerebrospinal fluid study [14].

**Figure 10:**
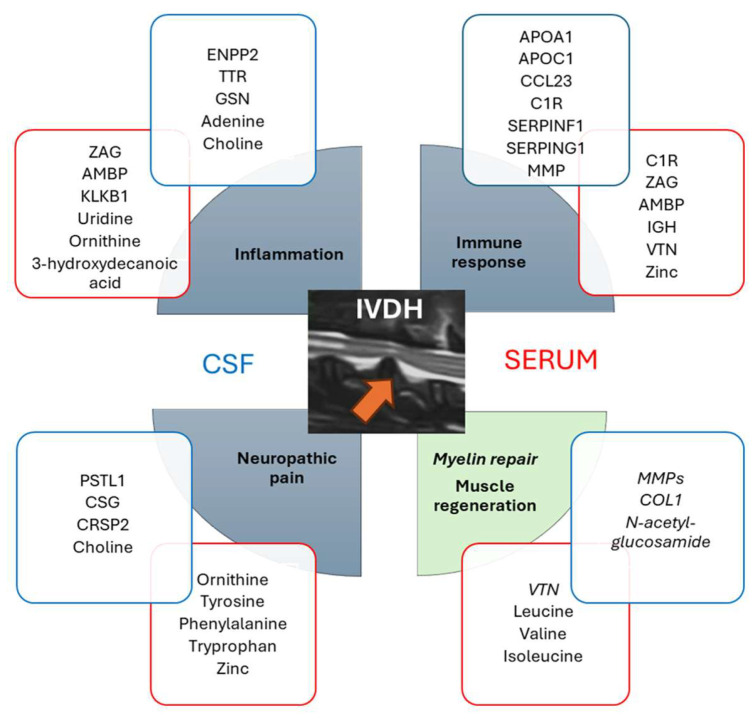
Processes and related proteins, metabolites, and trace element (Zn) observable in both serum (listed within red objects) and CSF (listed within blue objects) of dogs with IVDH.

The key molecules related to the abovementioned processes found by our group (Figure 10) and associated mechanisms were explained in detail in the previous sections. Interestingly, very few of the same deregulated molecules were found in both serum and CSF, indicating that the sample specificity was preferable for use in IVDH monitoring. Furthermore, analytical method specificities must also be considered, since no depletion of highly abundant proteins was performed, nor were other metabolomics analytical platforms used. Although CSF would be a better source of potential biomarkers as it is in direct contact with the central nervous system (CNS), the collection of this biofluid is rather invasive. Plasma or serum would be the ideal clinical samples, circumventing expensive and advanced diagnostic methods; however, serum biomarkers for IVDH have yet to be discovered.

Interestingly, the IVDH-related pathophysiological processes mentioned above could be monitored in tested biofluids (e.g., serum and CSF) at the molecular level; however, sample-specific molecular players were identified as deregulated (Figure 9). Although CSF provides a better understanding of processes related to CNS, serum proteomic and metabolomic profiles provide an overall picture of disease-related pathophysiological processes.

### 4.4. Strengths and Limitations of the Study

A major strength of our study is that the serum proteomes and metabolomes of healthy dogs and dogs with IVDH were analyzed and relatively quantified using high-resolution MS and subsequent data analysis. In addition, analytical validation provided absolute quantification results for selected proteins in serum, namely VTN, AMBP, and ZAG. The serum concentration of Zn was also determined and, to the authors’ best knowledge, provided information on the deregulation of this circulating trace element in relation to canine IVDH for the first time. The main limitation is the number of samples analyzed. In addition, dogs of different breeds, ages, and sexes were included in the study. However, this was a clinical study, and the remaining samples were used after the routine analysis required for the diagnosis and treatment of patients. It was also hard to collect suitable samples based on the population coming to our clinic, especially as older dogs are more prone to IVDH, and it was harder to find samples from healthy older dogs. However, taking into account all internal and external factors influencing the proteome and metabolome, the PLS-DA plot revealed a clear separation between healthy and IVDH dogs. The challenge for future research would be the collection of urine samples where collagen degradation products and zinc ions might be monitored to better understand the IVDH-related processes. Finally, clinical validation of selected metabolites and/or proteins is required to confirm their biomarker potential.

## 5. Conclusions

This study provides a comprehensive systemic characterization of canine intervertebral disc herniation in dogs presenting with acute paraplegia due to severe IVD herniation and nerve compression, through an integrative serum-based multi-omics approach. Our findings show significant metabolic and proteomic alterations, including disturbances in lipoic acid and branched-chain amino acid metabolism, implicating broader disturbances in energy regulation, nitrogen metabolism, and neurotransmitter signaling. The identification of elevated serum levels of ZAG, AMBP, and VTN, along with decreased levels of KLKB1 and SERPINA5, underscores the involvement of immunomodulation, neuroprotective responses, and coagulation pathways in the pathophysiology of IVDH. Furthermore, increased serum zinc concentrations highlight zinc’s critical antioxidant and immunomodulatory roles in neuropathic pain and tissue repair mechanisms. Pathway enrichment analyses revealed disturbances in aromatic amino acid biosynthesis, further linking neurotransmission to inflammatory processes to disease progression. Integration with previously obtained cerebrospinal fluid (CSF) multi-omics data strengthens the biological relevance of the identified molecular signatures, reinforcing their role in neurodegeneration, inflammation, and reparative processes associated with IVDH. Overall, these findings provide novel insights into the systemic molecular landscape of canine IVDH and identify potential serum biomarkers and therapeutic targets for improving the diagnosis, preoperative and postoperative care, and welfare of patients affected by IVDH.

## Figures and Tables

**Table 1:** Differentially abundant metabolites in the serum of dogs with IVDH compared to healthy dogs.

Peak ID	Metabolite	HMDB ID	Log2 FC	*p*-Value	Adj. *p*-Value
419	Ornithine *	HMDB0000214	1.00	0.0008	0.043
130	**Lysine**	HMDB0000182	0.82	0.0196	0.138
54	Isoleucine *	HMDB0000172	0.75	0.0002	0.032
39	Leucine *	HMDB0000687	0.61	0.0002	0.032
136	**Tyrosine**	HMDB0000158	0.56	0.0045	0.075
1341	**Tryptophan**	HMDB0000929	0.55	0.0099	0.104
147	Alpha-aminobutyric acid	HMDB0000452	0.51	0.0136	0.115
77	Phenylalanine *	HMDB0000159	0.50	0.0016	0.043
1213	Myristic acid	HMDB0000806	0.46	0.0398	0.199
35	Valine *	HMDB0000883	0.34	0.0003	0.032
1212	Citric acid	HMDB0000094	−0.21	0.0174	0.131
1551	Uridine	HMDB0000296	−0.26	0.0357	0.191
1326	**Creatinine**	HMDB0000562	−0.36	0.0034	0.064
1631	3-Hydroxydecanoic acid	HMDB0010725	−0.50	0.0438	0.211
126	Carnosine	HMDB0000033	−0.55	0.0219	0.144
486	Pipecolic acid	HMDB0000070	−0.60	0.0240	0.151
1307	N-acetyl glycine *	HMDB0000532	−0.67	0.0005	0.039
697	Spermine *	HMDB0001256	−0.75	0.0013	0.044
1636	**4-Hydroxyproline**	HMDB0000725	−0.96	0.0062	0.083

* Significant change after FDR correction; metabolites in bold identified using standard compound (Level 1); other metabolites annotated using fragmentation data (Level 2).

**Table 2:** Metabolic pathways (KEGG) altered in IVDH revealed in the serum of IVDH dogs based on deregulated metabolites. Arrows represent upregulated (↑) or downregulated (↓) pathways. The presented pathways were generated in MetaboAnalyst based on metabolite–metabolite interactions (related to Figure 2).

Pathway	Total	Hits	*p*-Value	Regulation
Lipoic acid metabolism	48	7	1.47 × 10^−9^	↑
Nitrogen metabolism	8	3	9.43 × 10^−6^	↑
Steroid hormone biosynthesis	4	2	0.000206	↑
Steroid biosynthesis	8	2	0.000948	↑
Oxidative phosphorylation	40	3	0.00150	↑
Fatty acid biosynthesis	36	2	0.00463	↓
Carbon metabolism	20	1	0.0405	↓

**Table 3:** The list of differentially abundant proteins in the serum of IVDH dogs compared to healthy dogs.

NCBI Accession ID	*p*-Value	log2FC	Gene Symbol	Protein Name	Number of Unique Peptides	MW/kDa	Calc pI
555289929	0.036	−3.64	KLKB1	prekallikrein	2	24	8.29
545508405	0.024	−1.64	SERPINA5	plasma serine protease inhibitor	7	45.8	8.44
124390007	0.048	−1.36	IGH	immunoglobulin heavy chain constant region CH1	3	11.5	4.83
545528321	0.036	−0.66	APOB	apolipoprotein B-100	42	518	6.95
296089	0.036	0.23	APOH	apolipoprotein H	13	38.4	8.13
70909945	0.036	0.67	AZGP1	zinc alpha-2-glycoprotein 1, partial	4	20.2	4.88
560879429	0.036	0.67	AZGP1	zinc-alpha-2-glycoprotein precursor	5	35.8	4.92
545504920	0.048	0.73	F5	coagulation factor V	2	251.7	5.77
1418267888	0.048	0.73	VTN	vitronectin	6	53.7	5.15
1239899336	0.048	0.80	C6	complement component C6	6	105.5	6.43
73997275	0.024	0.81	C1S	complement C1s subcomponent	5	77.5	4.93
163954	0.024	0.86	CLU	glycoprotein 80	13	51.8	5.91
57091057	0.048	0.88	AOC3	membrane primary amine oxidase isoform X1	4	84.2	6.37
1239918266	0.048	0.88	AOC3	membrane primary amine oxidase isoform X3	4	74.6	6.61
1418340066	0.048	0.88	AOC3	membrane primary amine oxidase isoform X2	4	84	6.25
1418515495	0.048	0.91	AHSG	alpha-2-HS-glycoprotein	7	39.2	5.31
1418256182	0.024	0.91	C1R	complement C1r subcomponent	9	80.1	6.24
345777712	0.048	1.05	AMBP	protein AMBP	2	38.8	6.35
1418324502	0.048	1.31	MGAM2	putative maltase-glucoamylase-like protein FLJ16351	2	282.1	5.38
1101972892	0.024	1.70	HBA1	globin A1	7	16.2	8.44
103484123	0.024	1.72	HBD	globin, partial	2	6.1	9.25
1418222276	0.024	1.75	HBB	hemoglobin subunit beta-like	6	12.8	6.68

**Table 4:** Reactome pathways altered in IVDH based on differentially abundant proteins related to the protein interaction network (Figure 5C) generated in Network Analyst. Arrows represent upregulated (↑) or downregulated (↓) pathways.

Pathway	Total	Hits	*p*-Value	Regulation
Platelet degranulation	89	2	0.00104	↑
Response to elevated platelet cytosolic Ca2+	94	2	0.00116	↑
Platelet activation, signaling, and aggregation	220	2	0.00621	↑
Scavenging of heme from plasma	15	1	0.00896	↑
Binding and uptake of ligands by scavenger receptors	15	1	0.00896	↑
Formation of fibrin clot (clotting cascade)	29	1	0.0173	↑
Molecules associated with elastic fibers	38	1	0.0226	↑
Elastic fiber formation	45	1	0.0267	↑
Hemostasis	511	2	0.0316	↑
Integrin cell surface interactions	85	1	0.0500	↑
LDL-mediated lipid transport	4	1	0.00060	↓
Chylomicron-mediated lipid transport	12	1	0.00180	↓
Platelet sensitization by LDL	19	1	0.00285	↓
Lipoprotein metabolism	22	1	0.00329	↓
Lipid digestion, mobilization, and transport	41	1	0.00614	↓
Retinoid metabolism and transport	41	1	0.00614	↓
Platelet homeostasis	88	1	0.0132	↓
Cell surface interactions at the vascular wall	99	1	0.0148	↓

**Table 5:** Concentrations of zinc alpha 2-glycoprotein (ZAG), al-pha-1-microglobulin/bikunin precursor (AMBP), and vitronectin (VTN) in the serum of healthy dogs and dogs with IVDH determined by ELISA.

Protein	Control Group Protein Concentration (Median/Range)	IVDH Group Protein Concentration (Median/Range)	*p*-Value *
zinc alpha 2-glycoprotein (ZAG)	37.97 ng/mL (13.55–43.41 ng/mL)	98.92 ng/mL (81.45–112.5 ng/mL)	0.0001
alpha-1-microglobulin/bikunin precursor (AMBP)	20.41 ng/mL (15.76–24.06 ng/mL)	33.08 ng/mL (26.44–41.8 ng/mL)	0.0004
vitronectin (VTN)	64.28 ng/mL (62.17–67.18 ng/mL)	78.84 ng/mL (74.39–95.45 ng/mL)	0.0031

* Assessed by Mann–Whitney U test.

## Data Availability

All results are presented within the manuscript and/or Supplementary Files. Proteomics mass spectrometry data are deposited in the MassIVE database available at https://massive.ucsd.edu/ProteoSAFe/static/massive.jsp?redirect=auth (accessed on 25 April 2025) (https://doi.org/10.25345/C5CC0V59N). Metabolomics mass spectrometry data will be made available upon request (anita.horvatic@pbf.unizg.hr).

## Supplementary Materials

The following supporting information can be downloaded at https://www.mdpi.com/article/10.3390/metabo15060396/s1, Supplementary File S1. List of identified and differentially abundant metabolites and affected metabolic pathways detected in the serum of dogs with IVDH compared to healthy dogs; Supplementary File S2. List of identified and differentially abundant proteins detected in the serum of dogs with IVDH compared to healthy dogs; Supplementary File S3. Metabolic pathways and reaction details involving the protein AOC3.

## Abbreviations

The following abbreviations are used in this manuscript:

AGC	Automatic gain control
AMBP	Alpha-1-microglobulin/bikunin precursor
AOC3	Amine oxidase copper containing 3
BCAA	Branched-chain amino acid
CSF	Cerebrospinal fluid
CNS	Central nervous system
CT	Computed tomography
DDA	Data-dependent acquisition
FDR	False discovery rate
GO	Gene Ontology
HCD	Higher-energy collision dissociation
IVD	Intervertebral disc
IVDH	Intervertebral disc herniation
KLKB1	Prekallikrein
MRI	Magnetic resonance imaging
NCE	Normalized collision energy
ROS	Reactive oxygen species
SERPINA5	Protein C inhibitor
TMT	Tandem mass tag
VTN	Vitronectin
ZAG	Zinc-α2-glycoprotein
